# A comparative analysis of using ensemble trees for botnet detection and classification in IoT

**DOI:** 10.1038/s41598-023-48681-6

**Published:** 2023-12-07

**Authors:** Mohamed Saied, Shawkat Guirguis, Magda Madbouly

**Affiliations:** Institute of Graduate Studies and Research, Alexandria, Egypt

**Keywords:** Engineering, Mathematics and computing

## Abstract

Enhancing IoT security is a corner stone for building trust in its technology and driving its growth. Limited resources and diversified nature of IoT devices make them vulnerable to attacks. Botnet attacks compromise the IoT systems and can pose significant security challenges. Numerous investigations have utilized machine learning and deep learning techniques to identify botnet attacks in IoT. However, achieving high detection accuracy with reasonable computational requirements is still a challenging research considering the particularity of IoT. This paper aims to analytically study the performance of the tree based machine learning in detecting botnet attacks for IoT ecosystems. Through an empirical study performed on a public botnet dataset of IoT environment, basic decision tree algorithm in addition to ensemble learning of different bagging and boosting algorithms are compared. The comparison covers two perspectives: IoT botnet detection capability and computational performance. Results demonstrated that the significant potential for the tree based ML algorithms in detecting network intrusions in IoT environments. The RF algorithm achieved the best performance for multi-class classification with accuracy rate of 0.999991. It achieved also the highest results in all other measures.

## Introduction

**Figure 1:**
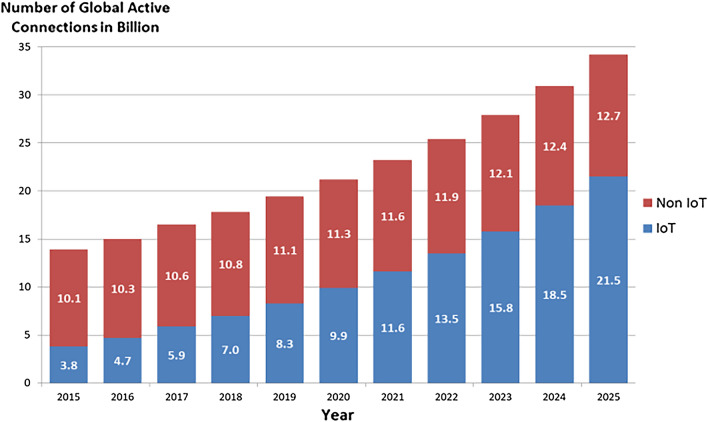
Number of global connected devices.

Internet of Things (IoT) technology enables the interconnectivity and communication of various objects for generating and exchanging data. The potential of IoT is vast as it has revolutionized many fields of our lives. It covers a wide range of applications including smart home, smart office, automated industry, smart city, smart agriculture, smart transportation system, supply chain, smart medical care, etc. Referring to IoT Analytics^1^, Fig. 1 illustrates the number of global active connected devices around the world. It shows an increasing growth of IoT devices compared to the non-IoT devices. It shows an annual increasing rate of 10% since 2018 and expected to reach 21.5 billion devices in 2025 exceeding three times the number of IoT devices of 2018. However, as IoT devices become more prevalent, security and confidentiality concerns have also raised.

IoT devices may be vulnerable to cyber-attacks due to its limited and heterogeneous configurations, which could lead to security threats. For example, in the Internet of Medical Things (IoMT)^2^, it may be required to transfer confidential patient’s health data to remote analytical devices. In case of lacking control, privacy leakage is probable due to network vulnerability for several types of attacks. Another motivating example is the connectivity of the industrial devices and sensors in the Industrial Internet of Things (IIoT). IIoT transformed the industry to another perspective which is the smart manufacturing. In order to automate the productivity and improve the availability, smart manufacturing requires connecting several devices such as smart sensors, robotics, and industrial servers. Resource constrained design of IIoT devices introduces several associated threats with the industrial systems^3^. Urban IoT environments are also facing security challenges considering the large-scale deployment of transportation infrastructures and their related sensor networks^4^. In addition, other geospatial security concerns for interconnecting transport systems with public infrastructures via IoT. Recent approaches tried to address these challenges through applying geospatial modeling in smart transportation security systems^4^, however those innovative approaches still have limitations.

Therefore, it is crucial to design and implement secure and robust IoT systems to ensure the availability, integrity and confidentiality of users’ data. To mitigate these security threats, IoT environment should be designed with security in mind. Intrusion detection is an essential part of network security, providing real-time protection against internal and external attacks. It involves detecting and responding to security threats in the IoT network. Network intrusion detection systems (NIDS) can be used to monitor network traffic and identify abnormal network traffic that may indicate a security breach or attack.

IoT botnet attack is a type of cyber-attack that targets IoT environment. It compromises IoT devices to be controlled remotely by attackers. It can be used to launch other types of cyber-attacks such as distributed denial of service (DDoS)^5^. These attacks can cause significant damage to the targeted IoT ecosystem, resulting in downtime, loss of revenue, or theft of sensitive data. Mirai is the name of the most botnet malware for IoT ecosystems. It caused several major websites to be disrupted by a series of massive DDoS attacks. The source code is designed to disrupt busy box systems and ultimately initiate very large-scale DDoS.

IoT botnet attacks are particularly dangerous because IoT devices often have weak security controls and are rarely updated with security patches. This makes them vulnerable to exploitation by attackers who can easily gain access to the devices and infect them with malware. Moreover, IoT devices are often connected to other devices and networks, which can amplify the impact of a botnet attack.

IoT botnet detection can be challenging due to the large number of diverse IoT devices and their heterogeneous configurations. While limited and constrained resources of IoT devices add further difficulties to an already challenging task^6^. Traditional intrusion detection techniques, such as signature-based detection and anomaly detection, may not be suitable due to the limited resources and the dynamic nature of IoT networks. In addition, different attacks have different signatures that makes feature selection a challenging task^7^.

To address these challenges, artificial intelligence techniques can present a great value. Through learning from the IoT network generated traffic and detect suspicious behaviors that may indicate an intrusion. To implement an effective IoT botnet detection system, both machine learning (ML) and deep learning (DL) can be used. ML can present benefits over DL for botnet detection in IoT. A number of approaches adopted DL models for botnet detection with comparable accuracy results^6^. However, the primary constraint is that it demands vast amounts of data to be trained effectively. ML requires less training data than DL to achieve good performance. This can be an advantage in IoT where data may be scarce or difficult to collect. In addition, it requires high computational demand that represents a limitation for IoT constrained nature that may lead to performance degradation with heavy network traffic^8^. To the contrary, ML provides relatively light alternative. It allows faster response time and often requires less computational power and resources than DL. In addition to its capability of being deployed across multiple IoT devices and networks. This makes ML more suitable for IoT environments.

Ensemble trees machine learning algorithms (ET) combine multiple decision trees to create a more accurate and robust model. This can lead to better prediction performance and lower error rates through reducing bias. ET algorithms are less sensitive to noise and outliers in the data. This makes them more robust and less prone to over fitting, which can improve the generalizability of the model. In the field of network intrusion detection, ET algorithms have shown great promise in effectively detecting and classifying network intrusions. ET algorithms offer several features that make them well-suited for intrusion detection in IoT networks. These algorithms combine multiple decision trees to create a robust ensemble model that can handle complex and high-dimensional data. The suitability of ET algorithms in IoT botnet detection scenarios stems from several factors.

- High dimensionality: IoT networks are characterized by a vast number of heterogeneous devices and sensors, generating a large volume of network traffic data. ET algorithms excel at handling high-dimensional data, as they can effectively capture complex relationships and interactions between features. This characteristic is particularly relevant in IoT environments where the number of features or attributes can be significant due to the diverse nature of IoT devices and the multitude of data sources.
- Non-linear relationships: IoT network traffic data often exhibits non-linear relationships and intricate patterns. ET algorithms, with their ability to capture non-linear interactions between features and hierarchical decision-making, are well-suited to model and detect these complex relationships. Unlike linear models, ET algorithms can capture non-linear decision boundaries, allowing for more accurate and flexible detection of network intrusions in IoT scenarios.
- Robustness to noisy data: In IoT environments, network traffic data can be noisy and prone to anomalies or outliers due to factors like sensor errors, communication interference, and varying environmental conditions. ET algorithms are inherently robust to noisy data due to their ensemble nature. By aggregating predictions from multiple decision trees, ensemble models can reduce the impact of individual noisy or mislabeled instances, leading to improved detection performance and resilience to data quality issues.
- Scalability: IoT networks can scale to include a massive number of devices, generating a vast amount of network traffic^9^. ET algorithms can handle scalable datasets efficiently, making them suitable for the large-scale nature of IoT environments. Additionally, ET algorithms can be parallelized, enabling distributed processing and scalability in IoT intrusion detection systems.
- Generalizability: ET algorithms are known for their robustness and ability to generalize well to unseen data. In the context of IoT networks, where the characteristics of devices and network conditions can vary significantly, it is crucial to have intrusion detection models that can adapt and generalize effectively. Through their ensemble averaging and voting mechanisms, can reduce the risk of overfitting and provide reliable and accurate detection results.
- Computational efficiency: ET algorithms are computational efficient for real-time or near real-time detection, which is crucial in rapidly evolving IoT environments.

Bagging and boosting are two main types of ensemble learning methods. The key difference between them relies on the training way or how the trees are built and combined. Bagging adopts parallel training, while boosting adopts sequential learning. This paper conducts a comprehensive comparative study of multiple ET models i.e. Decision Trees (DT)^10^, Random Forest (RF)^11^, Bagging Meta Classifier (BMC)^12^, Adaptive Boosting (ADB)^13^, Gradient Descent Boosting (GDB)^14^, and eXtreme Gradient Boosting (XGB)^15^. Both BMC and RF use bagging technique to build full DT in parallel. The difference relies in the prediction method. RF implies averaging for the final output, while BMC implies linear voting combination. Gradient boosting extends the concept of boosting by utilizing a gradient descent algorithm to iteratively generate a series of weak models. The final prediction is computed by combining the predictions of all the weak models, with each model being assigned a weight determined by its performance during training. Specifically, DT is often employed as the base learners in gradient boosting, and each new tree is trained to predict the residuals of the previous model. XGB is an implementation of Gradient Boosted Decision Trees^16^. It uses boosting technique that aggregates all predictions from its constituent learners in a sequential manner. In such way, each tree eliminates the error of its previous trees to update the residual error. For models learning and evaluation, this study employs an IoT environment based dataset N-BaIoT^17^.

The main contributions of this paper can be listed as follows.

1. Examining the literature of using ensemble trees algorithms in IoT network intrusion detection.
2. Presenting a comprehensive efficient botnet detection model for IoT ecosystems with detailed preprocessing operations for multi-class classification.
3. Conducting an exploratory data analysis (EDA) for N-BaIoT dataset ^17^ to analyze and summarize their main characteristics and features.
4. Investigating the potential of ET methods for detecting IoT botnet attacks through an experimental performance evaluation of six ML tree-based algorithms representing basic decision tree (DT), bagging technique based algorithms (RF, BMC) and boosting technique based algorithms (ADB, GDB, XGB).
5. Benchmarking the six models through a computational analysis to gain more insight into how light they are to an IoT environment.
6. Validating the best performance model using fivefold cross validation and ensuring its generalization capability through analyzing its learning curve through its training score and the cross-validation score.
7. Comparing best performance results with a deep learning based intrusion detection approach from the literature ^18^.

The rest of this paper is organized as follows. Section 2 provides an overview of related work. Section 3 presents a research gap analysis of related work. Section 4 describes the dataset used. Section 5 demonstrates the empirical investigation procedure and Section 6 presents the evaluation metrics, and conducts an extensive empirical study for comparing the pre-mentioned ET-based algorithms. It reports the outcomes and performance trajectory in addition to a computational evaluation. Section 7 concludes this work.

## Related work

Numerous studies adopted DT algorithm for detecting network intrusions in IoT. For instance, Bahsi et al.^19^ utilized feature selection methods to reduce the number of features and enhance accuracy. The authors evaluated two ML classification algorithms, namely DT and k-Nearest Neighbors (kNN), and found that the accuracy of kNN was 94.97%, which was lower than that of DT 98.97%. The researchers simulated an IoT network consisting of nine IoT devices, including a baby monitor, thermostat, doorbell, security camera, and webcam. They labeled their dataset with three labels, namely normal, Bashlite, and Mirai, which contained 502,605 normal records, 2,835,317 Bashlite records, and 2,935,131 Mirai records.

Aloqaily et al.^20^ utilized deep belief and DT mechanisms to detect intrusions in an internet of vehicles environments (IoV). Their proposed model attained an accuracy of 99.43% using a simulated dataset consisting of 22,544 records.

Anthi et al.^21^ proposed a supervised approach consisting of three layers for detecting and classifying intrusions in IoT. The system performs three main functions: creating a normal behavior profile for each IoT device, identifying malicious packets in case of an attack, and classifying the type of attack. To evaluate their approach, they built a smart home test bed comprising eight IoT devices and injected 12 attacks categorized into four main types, namely man in the middle (MITM), denial of service (DoS), reconnaissance, and replay. They selected nine classifiers, including Naive Bayes (NB), Bayesian Network, Java implemented DT (J48), Zero R, One R, Simple Logistic, Support Vector Machine (SVM), Multi-Layer Perceptron (MLP), and RF, based on their ability to support multi-class classification, classification time, and high-dimensional feature space. The results indicated that the DT J48 model achieved the best performance, with reported evaluation results of 96.2% for device profiling, 90.0% for detecting wireless attacks, and 98.0% for attack type classification.

Goyal et al.^22^ proposed a behavioral analysis-based approach for botnet detection and evaluated Logistic Regression (LR), SVM, Artificial Neural Network (ANN), and DT. They reported accuracy rates of 99.23%, 99.86%, and 99.74% for LR, SVM, and ANN, respectively, while no accuracy was reported for DT.

Illy et al. ^23^ utilized ensemble classifiers and combined different ML algorithms for intrusion detection, using DT Bagging Ensemble technique DT (BE) on the NSL-KDD dataset^24^. They achieved accuracies of 85.81% and 84.25% for binary and attack classifications, respectively.

Alsulami et al.^25^ investigated several ML algorithms for intrusion detection in IoT with IoTID20 dataset^26^. The algorithms were ANN, DT, Bagged Trees (BT), SVM, and kNN. The classification accuracy results were reported as 100% for ANN, DT, and BT, while 99.80% and 99.40% for KNN and SVM, respectively.

Chaudhary and Gupta^27^ proposed an ML-based framework for detecting DDoS attacks in two phases, namely detection and mitigation. They collected a dataset by capturing traffic from an IoT environment consisting of PCs and Raspberry Pi devices using Wireshark, which contained a total of 114,565 packets, including 10,061 benign packets. To classify the data, they evaluated four algorithms, namely RF, SVM, LR, and DT, and reported accuracy rates of 99.17, 98.06, 97.50, and 98.34, respectively.

Another research area has focused on the adoption of the RF algorithm for network intrusion detection in IoT. Manimurugan et al.^28^ proposed a deep belief model for intrusion detection in smart medical environments, using the CICNIDS 2017 dataset^29^. Their model achieved a good accuracy rate of 99.37 for the benign class; but unsatisfactory accuracy for anomalies. The highest detection accuracy was for Web attacks with 98.37, while both brute brute force and port scans were detected with a rate of 97.71. The least accuracy rates were for Dos/DDoS with 96.67 and Infiltration with 96.37.

Alsamiri and Alsubhi^30^ assessed the performance of seven ML algorithms for detecting IoT network attacks using the Bot-IoT dataset^31^. They reported the highest detection accuracy rate of 99% for kNN, while RF, Iterative Dichotomiser3 (ID3), and ADB achieved lower performance with accuracy rates of 97%. Quadratic Discriminant Analysis (QDA), MLP, and NB achieved unsatisfactory accuracy rates of 87%, 84%, and 79%, respectively.

Doshi et al.^32^ created a labeled training dataset by simulating a local network of consumer IoT devices, which included both benign and malicious traffic. They used this labeled dataset to evaluate five different ML classifiers: KNN, SVM with linear kernel (LSVM), ANN with four-layer fully-connected feed-forward architecture, DT, and RF using Gini impurity scores. The researchers reported that the inclusion of stateful features led to higher accuracy compared to using stateless features alone. RF achieved the highest accuracy of 99.8%, outperforming KNN and DT which achieved 99.5% accuracy and ANN which achieved 98.9% accuracy. On the other hand, LSVM had the worst accuracy of 92.1%.

Dwyer et al.^33^ proposed a Domain Name Service (DNS) based profiling technique to identify Mirai-like botnet activities. Their approach relies on analyzing the contents of DNS queries and using RF classifier for classification. They tested their approach on real honeypot datasets and compared it with Bayesian-based classifiers and kNN. The RF classifier achieved the highest accuracy of 99%.

Hasan et al.^34^ conducted a study to compare different algorithms for detecting and classifying intrusions in IoT, including LR, SVM, DT, RF, and ANN. They utilized the Pahl open source dataset^35^, which contains synthetic data from the Distributed Smart Space Orchestration System (DS2OS) IoT environment. The researchers found that RF had the best accuracy performance, achieving 99.4%. However, their study was limited to a specific dataset and did not address issues related to big data or unknown problems.

Chaudhary and Gupta^27^ proposed a ML framework for detecting DDoS attacks, which operates in two phases: Detection and Mitigation. They collected a dataset by capturing traffic from an IoT environment consisting of personal computers and Raspberry Pi devices, using Wireshark. The dataset contained a total of 114,565 packets, with 10,061 of them being benign. The researchers evaluated four algorithms, namely RF, SVM, LR, and DT for classification and reported accuracy rates of 99.17%, 98.06%, 97.50%, and 98.34%, respectively.

Alrashdi et al.^36^ suggested an NIDS for IoT in a smart city using the RF and Extra Tree. They evaluated their model using the UNSW-NB15 dataset^37^ and reported a detection accuracy of 99.34% with the lowest false positive rate.

Thamilarasu et al.^38^ presented a mobile agent-based intrusion detection system for medical IoT and simulated a hospital network topology for the Internet of Medical Things. They trained five supervised ML algorithms, including SVM, DT, NB, KNN, and RF. The researchers reported unsatisfactory performance for KNN and NB, while SVM, DT, and RF performed well. Among the algorithms, RF superseded with an approximated accuracy of 100%.

Eskandari et al.^39^ introduced an intelligent NIDS for IoT using lightweight one-class classification ML algorithms called Passban. Their approach utilized two one-class classification techniques, namely Isolation Forest (iForest) and Local Outlier Factor (LOF). The researchers created an IoT test bed to mimic a typical smart home automation environment and evaluated their approach in two scenarios: deploying NIDS directly on the IoT gateway and using a separate independent NIDS. They tested the NIDS against four different attacks, including port scanning, HTTP brute force, SSH brute force, and SYN flood attack, and reported detection accuracy rates ranging from 79% to 99%.

Hammoudeh and Aljaberi^40^ proposed intrusion detection system for IoT based on the gated recurrent unit (GRU) deep learning algorithm with flower pollination algorithm (FPA) for feature selection with an accuracy of 99.59%. They conducted an extensive experimental analysis for evaluating their approach against some ML based models i.e. DT, RF, LR, and an ensemble of several ML algorithms (SVM + DT + RF + LR + GDB). The accuracy rates were reported as 91.04, 89.39, 90.37, and 92.03, respectively. Their study was employed on KDD Cup 99 dataset^41^. However, KDD Cup 99 is an old dataset and does not address IoT network intrusions. It does not include HTTP DoS or botnet attacks.

Saied and Guirguis conducted an evaluative study for evaluating the performance of tree based approaches for intrusion binary detection in IoT^42^. They utilized the N-BaIoT dataset^17^ for models training and testing. They compared six different algorithms for binary detection (DT, RF, Bagging Meta Classifier (BMC), Adaptive Boost model (ADB), Gradient Descent Boosting (GDB), and Extreme Gradient Boosting (XGB). They reported the best performance using RF based model with a detection accuracy rate of 99.99%.

Only a few studies have utilized boosting techniques for network intrusion detection in IoT. Saied et al.^43^ presented a comparative study for boosting based algorithms in detecting intrusions in IoT. Their study benchmarked the performance of six boosting based algorithms in multi-class classification. Those algorithms are Adaptive Boosting (ADB), Gradient Descent Boosting (GDB), Extreme Gradient Boosting (XGB), Categorical Boosting (CAB), Hist Gradient Boosting (HGB), and Light Gradient Boosting (LGB). Their study utilized N-BaIoT dataset^17^ through 115 selected features. They reported that HGB outperformed with 99.99% of detection accutacy.

Alqahtani et al.^44^ proposed an approach that utilizes the XGB algorithm for detecting intrusions in IoT. They reduced the number of features in the N-BaIoT dataset^17, 45^ and achieved an accuracy rate of 99.97% in multi-class classification. Qasem et al.^46^ employed ADB algorithm combined with DT for detecting cyber-attacks in IoT networks. They evaluated their model on the TON_IoT dataset^47^ and reported an overall accuracy of 99.70 for multiclass classification of nine types of attacks: injection, password, ransomware, backdoor, scanning, MITM, DoS, DDoS, and XSS. Al-Haija et al.^48^ proposed an ensemble learning model for botnet attack detection in IoT. Their approach is to applying the voting based probability to ensemble the three ML classifiers i.e. ADB, Random under sampling boosting model (RUS), and bagged model. The individual performance for the selected classifiers was 97.30, 97.70, and 96.20, respectively. The performance of the proposed ensemble model was 99.60%.

Table 1 presents a comparative analysis for the previous related work in tabular form. The table shows how the majority of presented methods were tested using simulated datasets. A few of them have been tested using standard well known datasets (i.e. NSL-KDD^49^, CICNIDS^50^, and UNSW-NB15^37^). It leads to missing a unique datum for performance evaluation of the proposed approaches. As each of them depends on different simulated dataset, the reported high accuracy values cannot be considered in benchmarking with other approaches.

**Table 1:** Comparative analysis for the related work.

Algorithm	Author	References	Year	Dataset	Objective	No of classes	No of features	Accuracy
DT	Bahsi	^19^	2018	Simulated	Reduce dimensionality of ML based IoT botnet detection	3	10	98.97
DT	Aloqaily	^20^	2019	NSL-KDD	Intrusion detection in connected vehicles	5	122	99.43
DT (J48)	Anthi	^21^	2019	Simulated	Intrusion detection in smart medical IoT	2	121	99.00
DT (J48)	Anthi	^21^	2019	Simulated	Intrusion detection in smart medical IoT	4	121	98.00
DT	Goyal	^22^	2019	Simulated	Detecting botnets based on behavioral analysis in IoT	2	3	87.15
DT (BE)	Illy	^23^	2019	NSL-KDD	Securing Fog-to-Things	5	38	85.81
DT (BE)	Alsulami	^25^	2020	IoTID20	Intrusion detection and classifying in IoT	5	71	100%
DT	Chaudhary	^27^	2019	Simulated	DDoS detection in IoT	2	NA	98.34
RF	Chaudhary	^27^	2019	Simulated	DDoS detection in IoT	2	NA	99.17
DT	Manimurugan	^28^	2020	CICNIDS	Intrusion detection in smart medical IoT	6	80	98.37
RF	Doshi	^32^	2017	Simulated	DDoS detection in IoT	2	11	99.80
RF	Dwyer	^33^	2018	Real Dataset	Profiling IoT botnet traffic using DNS	5	6	99.00
RF	Hasan	^34^	2019	Pahl	Intrusion detection and classifying in IoT	8	13	99.40
RF + ET	Alrashdi	^36^	2019	UNSW-NB15	NIDS for IoT	2	49	99.34
RF	Thamilarasu	^38^	2020	Simulated	Intrusion detection for medical IoT	2	NA	100.0
RF	Eskandari	^39^	2020	Simulated	NIDS for IoT	5	24	99.00
RF	Hammoudeh	^40^	2021	KDDCup99	NIDS for IoT	2	41	89.39
RF	Saied	^42^	2023	N-BaIoT	NIDS for IoT	2	115	99.99
ADB	Qasem	^46^	2021	TON_IoT	NIDS for IoT	9	NA	99.70
ADB	Al-Haija	^48^	2022	N-BaIoT	Botnet attack detection in IoT	3	10	97.30
RUS								97.70
ELBA								99.60
HGB	Saied	^43^	2023	N-BaIoT	Botnet attack detection in IoT	3	115	99.99
XGB	Alqahtani	^44^	2020	N-BaIoT	IoT Botnet Attack Detection	3	3	99.96

## Methods

The objective of knowledge extraction from data such as network intrusion detection is made possible by ML through a mechanism known as Machine Learning Life Cycle^51^. This section introduces the proposed comparative scheme and the selected dataset in the context of the ML life cycle. It describes the overall design of the comparative scheme, including the dataset used and the machine learning algorithms employed.

### Proposed scheme

**Figure 2:**
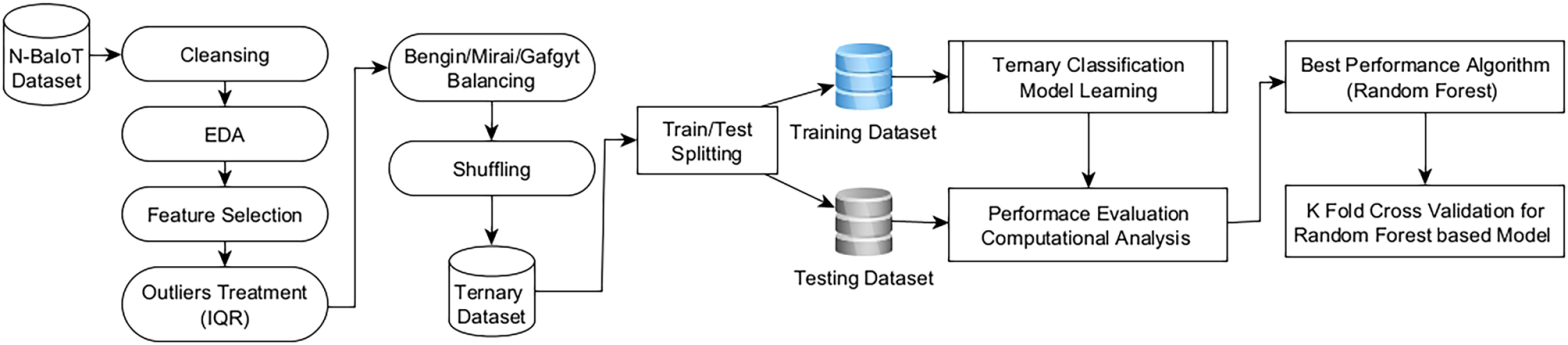
Proposed evaluation scheme.

Figure 2 illustrates the proposed scheme for models evaluation. There are four primary stages involved in this scheme. The first stage is the dataset preprocessing. It is an essential step in preparing data for machine learning algorithms. It involves cleaning, transforming, and reformatting the raw data to make it suitable for use in a machine learning model. The quality of the preprocessing step can have a significant impact on the performance of the machine learning algorithm. The second stage involves dataset balancing, shuffling and splitting into two subsets: a training set and a test set with 80/20 ratio. The third stage is model learning and evaluation. The training set is used to train the model, while the test set is used to evaluate the performance of the model on new, unseen data. The test set is used to estimate the generalization error of the model, which is the error rate that the model is expected to achieve on new data.

This study considers six tree based algorithms for empirical evaluation, which are DT, RF, Bagging Meta Classifier (BMC), ADB, Gradient Descent Boosting (GDB), and XGB. The experiments are conducted using Colab notebook interactive environment.

### Dataset selection

There are many network traffic extracted datasets available on the internet. In this study, N-BaIoT^17^ dataset is selected for training and evaluation purposes. The selection of the N-BaIoT dataset in the study has several benefits. First, the dataset is collected from an IoT environment, which is relevant to the study's focus on IoT security. Second, the dataset includes injected malicious traffic of botnet attacks, which provides a realistic scenario that can help evaluate the effectiveness of the selected ML algorithms in detecting and mitigating botnet attacks. Third, the dataset is gathered from multiple sources including telemetry, operating systems, and network sources, which provides a diverse set of data that can help improve the accuracy and robustness of the proposed method. Fourth, the N-BaIoT dataset is widely accepted as a benchmark sequential dataset, which facilitates the comparison of the proposed methods' performance with other state-of-the-art methods. Finally, the N-BaIoT dataset contains realistic network traffic and a variety of attack traffic, which can help enhance the generalizability of the proposed method to other IoT environments and attack scenarios.

### Dataset description

This section describes the dataset used in experiments. It was suggested by Meidan et al.^17^ through gathering traffic of nine commercially available IoT devices authentically infected by Mirai and Bashlite malware. The devices were two smart doorbells, one smart thermostat, one smart baby monitor, four security cameras and one webcam. Traffic was captured when the devices were in normal execution and after infection with malware.

The malware infection includes ten attack types of DDoS.

1. Mirai_Ack: A variant of Mirai botnet that uses Acknowledge flooding (ACK) to carry out DDoS. In an ACK flood attack, an attacker sends a large number of ACK packets to a target system, overwhelming its ability to process legitimate traffic.
2. Mirai_Scan: A variant of Mirai botnet that uses scanning methods to identify vulnerable devices for recruitment into the botnet.
3. Mirai_Syn: A variant of Mirai botnet that uses Synchronize flooding (SYN) to carry out DDoS. In a SYN flood attack, an attacker sends a large number of SYN requests to a target system without completing the three-way handshake. This can cause the system's resources to become exhausted as it waits for the handshake to complete.
4. Mirai_UDP: A variant of Mirai botnet that uses User Datagram Protocol (UDP) flooding to carry out DDoS.
5. Mirai_UDPPlain: A variant of Mirai botnet that uses User Datagram Protocol (UDP) flooding and optimized for higher packets rate.
6. Gafgyt_Combo: A variant of the Gafgyt botnet that uses a combination of DDoS attack methods simultaneously to overwhelm the target system. For example, an attacker may use a combination of SYN flooding, UDP flooding, and TCP flooding to make it more difficult for the target system to defend against the attack.
7. Gafgyt_Junk: A variant of the Gafgyt botnet that uses junk traffic to carry out DDoS.
8. Gafgyt_Scan: A variant of the Gafgyt botnet that uses scanning methods to identify vulnerable devices for recruitment into the botnet.
9. Gafgyt_TCP: A variant of the Gafgyt botnet that uses TCP flooding to carry out DDoS. In a TCP flood attack, an attacker sends a large number of TCP packets to a target system, which can cause it to become overwhelmed and unresponsive.
10. Gafgyt_UDP: A variant of the Gafgyt botnet that uses UDP flooding to carry out DDoS. In a UDP flood attack, an attacker sends a large number of UDP packets to a target system, which can cause it to become overwhelmed and unresponsive.

The traffic was captured through network sniffing utility into raw network traffic pcap format. It can be achieved through using port mirroring. Five features are extracted from the network traffic as abstracted in Table 2. Three or more statistical measures are computed for each of these five features for data aggregation, resulting in a total of 23 features. These 23 distinct features are computed over five separate time-windows (100 ms; 500 ms; 1.5 s; 10 s; and 1 min). Using time windows makes this dataset appropriate for stateful IDS and resulting in total of 115 features.

**Table 2:** Dataset attributes information.

Stream aggregation designation	Stream aggregation description	Stream characteristics (statistical aggregation functions)							Count	Time frame	Features
		Weight	Mean	Variance/standard deviation	Magnitude	Radius	Covariance	Correlation coefficient			
H	Host Source IP	✓	✓	Variance	X	x	x	x	3	5	15
MI	Host Source IP + MAC	✓	✓	Variance	x	x	x	x	3	5	15
HH	Host to Host channel (Source IP to destination IP)	✓	✓	Std	✓	✓	✓	✓	7	5	35
HH_Jit	Host to Host channel jitter	✓	✓	Variance	x	x	x	x	3	5	15
HpHp	Host port to Host port channel (IP + Socket)	✓	✓	✓	✓	✓	✓	✓	7	5	35
								Tot. traffic characteristics	23	Tot. features	115

**Figure 3:**
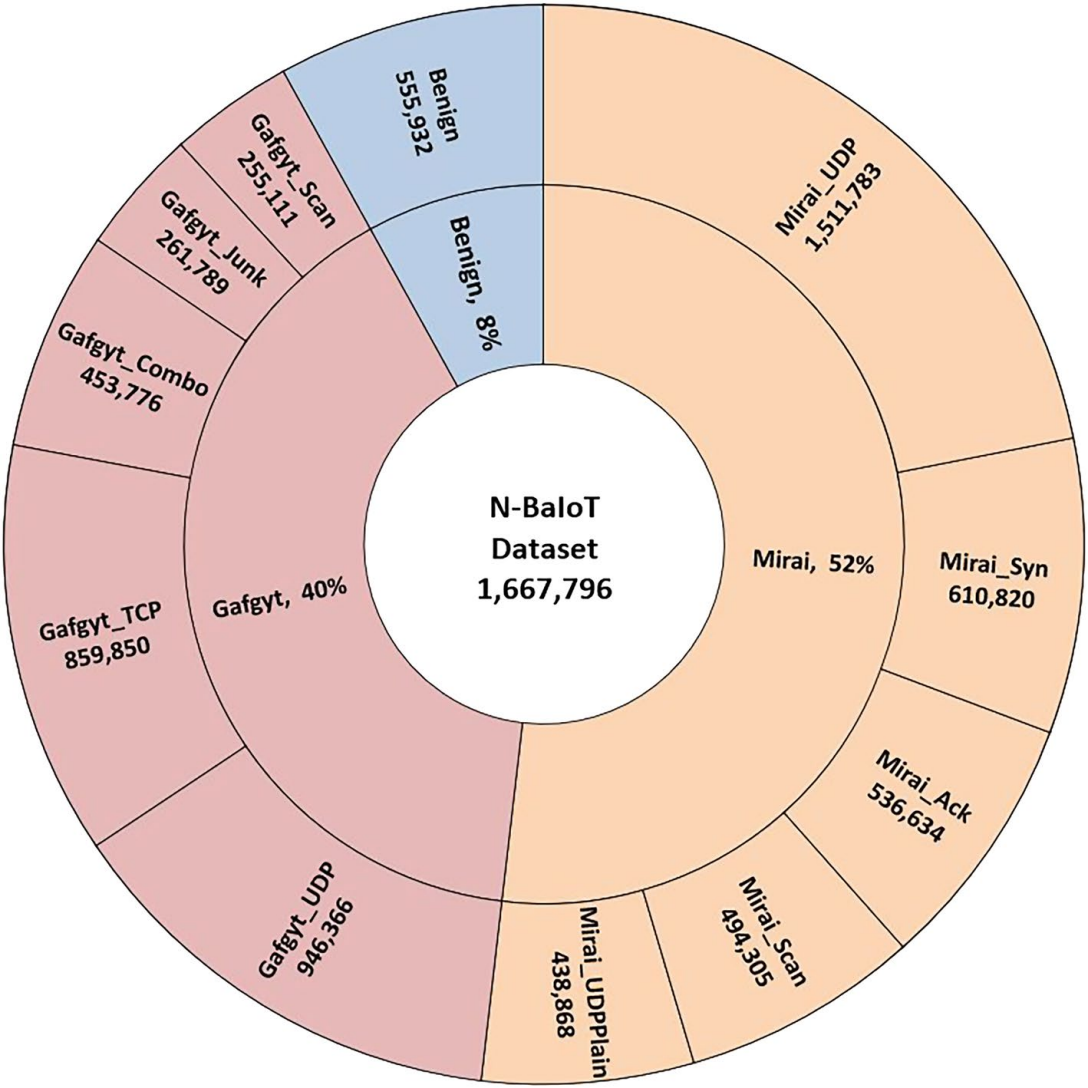
Dataset exploration.

The dataset contains instances of network traffic data divided into three categories: normal traffic (Benign), Bashlite infected traffic, and Mirai infected traffic. Each data instance consists of 115 features represented by 23 different traffic characteristics in five different time frames. Table 2 presents an abstracted demonstration for the dataset attributes information. Figure 3 shows the data exploration in a radial tree map (Sunburst graph) for the dataset collected. The innermost ring represents the distribution of the three labeled types i.e. benign, Mirai and Gafgyt. With each subsequent outer ring, the subcategories represent the related dataset individual distribution of the 10 malware classes in addition to the benign traffic.

### Dataset preprocessing

As shown in Fig. 3, N-BaIoT^17^ dataset is unbalanced. Therefore, a subset of it was selected to form balanced multi-class labeled dataset. All benign traffic is considered containing 555,932 instances. The rest malicious traffic datasets are merged into two collective subsets i.e. Mirai and Gafgyt.

Mirai category includes (Mirai_Ack, Mirai_Scan, Mirai_Syn, Mirai_UDP, and Mirai_UDPPlain). Gafgyt category includes (Gafgyt_Combo, Gafgyt_Junk, Gafgyt_Scan, Gafgyt_TCP, and Gafgyt_UDP). Each category was labeled accordingly. In order to have a balanced dataset, same number of benign instances is selected from each malicious category. In such way, the total number of instances is equal for each class of the three representing a balanced dataset of total 1,667,796 instances as shown in Table 3.

**Table 3:** Dataset balancing.

Dataset	Classifier	Class	Training set	Testing set
1,667,796	Benign	555,932	1,334,237	333,559
	Mirai	555,932		
	Gafgyt	555,932		

The dataset is then randomly shuffled to randomize the order of the training data before feeding it into the learning algorithms. The purpose of shuffling is to prevent any patterns in the data from affecting the order in which algorithm learns.

### Feature distribution and reduction

**Figure 4:**
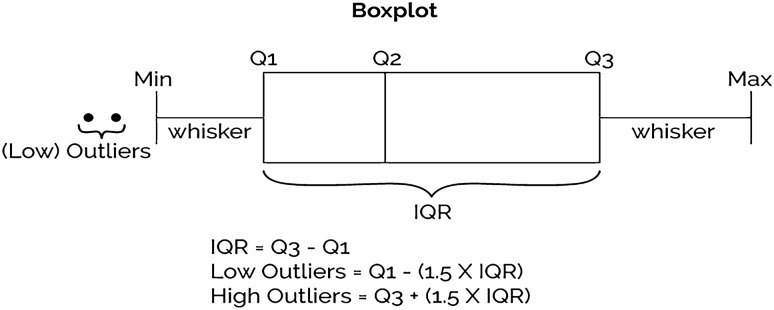
Interquartile range and boxplot.

**Figure 5:**
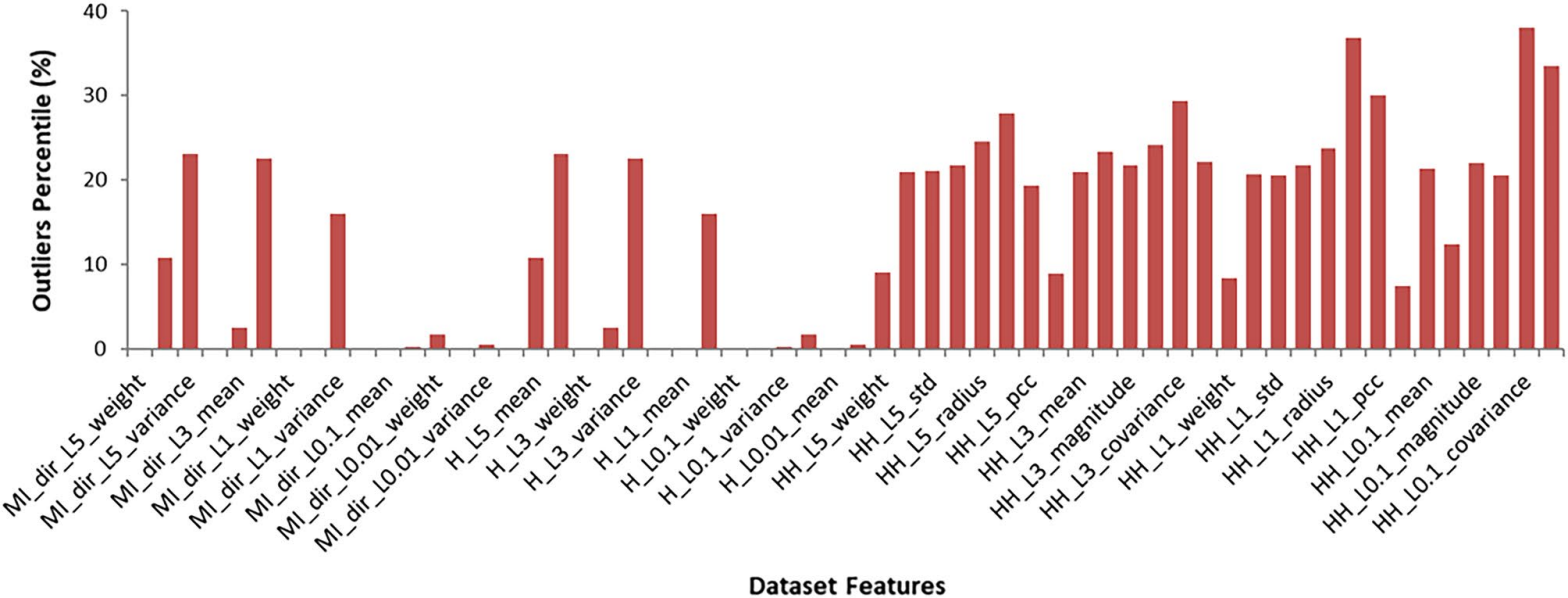
Features outliers percentile.

Feature distribution is the term used to describe the spread or range of values that a particular feature or variable takes on within a dataset. Understanding the feature distribution can help to identify potential outliers, anomalies, or errors in the data. It can inform decisions about data cleaning, scaling, or normalization. Interquartile Range (IQR) is a technique used to identify and remove potential outliers from a dataset. It is calculated as the difference between the 75th percentile (Q3) and the 25th percentile (Q1) of the distribution as shown in Fig. 4. In this study, IQR is used for analyzing the dataset’s features. In order to calculate the percentage of the outliers in each feature, IQR is calculated, then calculating the percentage of the falls below Q1 − 1.5 × IQR or above Q3 + 1.5 × IQ. Figure 5 illustrates the outliers’ percentile for all dataset features. As outliers can have a disproportionate impact on the feature distribution, and can lead to overfitting or biased models, they are replaced by the mean value of the related feature.

### Evaluation metrics

The confusion matrix is used to visualize the performance of a technique. It is a table that is often used to describe the performance of a classification model on a set of test data. It allows easy identification of confusion between classes. They are used to calculate other performance measures. The classification is evaluated through four indicators:

- True positives (TP): packets are predicted as malicious, and their ground truth is malicious.
- True negatives (TN): packets are predicted as benign, and their ground truth is benign.
- False positives (FP): packets are predicted as malicious, while their ground truth is benign.
- False negatives (FN): packets are predicted as benign, while their ground truth is malicious.

A successful detection requires correct attacks identification with minimizing the number of false alarms.

In order to perform a comprehensive performance assessment and objective evaluation, several metrics shall be addressed to indicate how model performs. Accuracy only is not sufficient for imbalanced dataset.

Four metrics are widely used for evaluating ML models i.e. accuracy, precision, recall, F1 score and specificity. Those four measures are defined through the following equations respectively. The goal is to maximize all measures, which range from 0 to 1. The higher values correspond to better classification performance.

$$Accuracy= \frac{TP+TN}{TP+TN+FP+FN}$$

$$Precision= \frac{TP}{TP+FP}$$

$$Detection Rate= Recall (Sensitivity)= \frac{TP}{TP+FN}$$

$$F1 Score= \frac{2 \times Precision \times Recall}{Precision+Recall}$$

## Results

This section explains the confusion matrix and evaluation metrics used for comparing. It presents the experimental. Then, discussion for results is conducted.

In this empirical study, the six tree based algorithms are evaluated for the objective of multi-class classification for the network traffic. The evaluation metrics are calculated and documented in Table 4. The empirical evaluation results showed significant potential for the ensemble tree based ML algorithms in detecting network intrusions in IoT.

**Table 4:** Evaluation results for multi-class classification.

Technique	Classifier	Class	Accuracy	Precision	Detection rate	F1 score	Training time (s)	Testing time (s)	Detection time (μs)
DT	DT	Benign	0.999973	0.999955	0.999973	0.999990	78.38	0.09	0.269
		Mirai		0.999973	0.999973	0.999972			
		Gafgyt		0.999964	0.999973	0.999981			
Bagging	BMC	Benign	0.999979	0.999964	0.999982	0.999990	5919.21	16.59	49.736
		Mirai		0.999982	0.999973	0.999981			
		Gafgyt		0.999973	0.999977	0.999986			
	**RF**	**Benign**	**0.999991**	**0.999973**	**1.000000**	**1.000000**	**685.47**	**3.19**	9.563
		**Mirai**		**1.000000**	**0.999982**	**0.999990**			
		**Gafgyt**		**0.999986**	**0.999991**	**0.999995**			
Boosting	ADB	Benign	0.952566	0.999028	0.876440	0.999979	1645.29	9.54	28.600
		Mirai		0.998830	0.999309	0.859125			
		Gafgyt		0.998929	0.933850	0.924216			
	GDB	Benign	0.9998890	0.999730	0.999937	1.000000	10,125.00	3.47	10.402
		Mirai		0.999946	0.999739	0.999981			
		Gafgyt		0.999838	0.999838	0.999990			
	XGB	Benign	0.9994004	0.999289	0.999085	0.999828	2157.26	2.16	6.475
		Mirai		0.999136	0.999157	0.999909			
		Gafgyt		0.999213	0.999121	0.999869			

Significant values are in bold.

In the context of intrusion detection, F1 score can be used to evaluate the overall performance of the model in detecting both true positives and avoiding false positives. A high F1 score indicates that the model is effectively detecting intrusions while minimizing false alarms. Recall is an important measure in the context of intrusion detection, as it indicates the model's ability to correctly identify all instances of intrusions. A high recall score indicates that the model is effectively detecting all instances of intrusions, even if it means including some false positives. This is particularly important in the context of intrusion detection, as missing even a single intrusion can have serious consequences.

**Figure 6:**
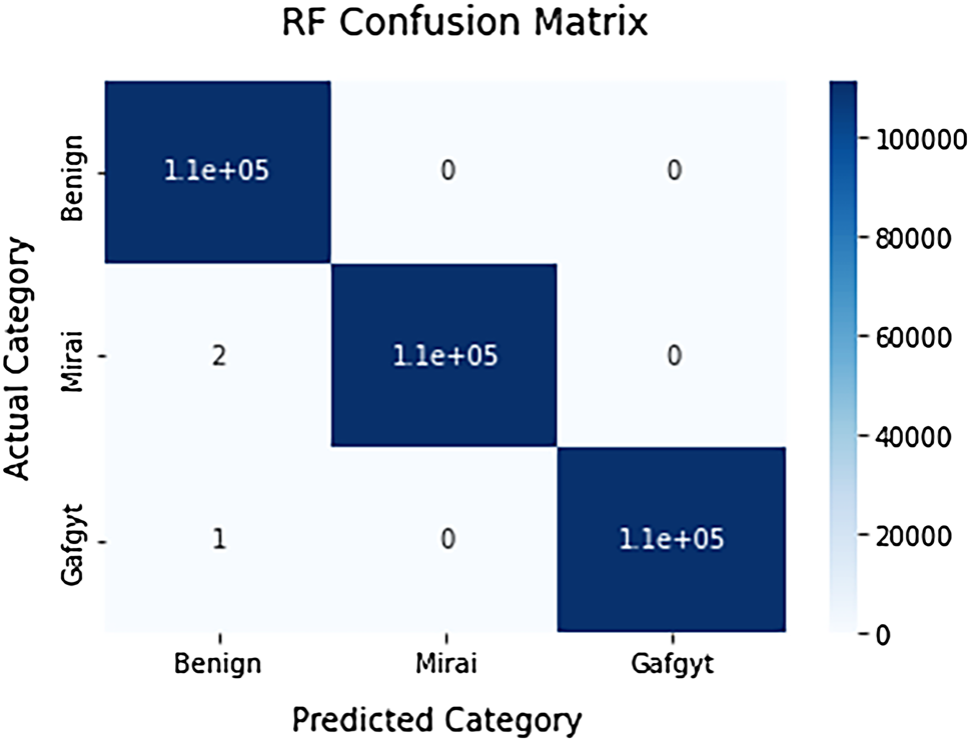
Confusion matrix for RF multi-class classifier.

Precision is the ratio of true positive predictions to the total number of positive predictions and measures the model's ability to avoid false alarms. A high precision score indicates that the model is effectively detecting intrusions while minimizing false alarms. The RF algorithm achieved the best performance for multi-class classification with accuracy rate of 0.999991. It achieved also the highest results in all other measures. Figure 6 shows its confusion matrix of multi-class classification.

Regarding the training time, GDB algorithm spent the longest training time. It is because GDB does not support multi-threading. Unlike the XGB algorithm that is an implementation of GDB supporting multithreading.

To ensure the robustness and dependability of our findings, we incorporated cross-validation as a crucial step in our research methodology. Cross-validation is a widely recognized technique utilized to evaluate the predictive model's generalization performance. In our study, we implemented k-fold cross-validation, which involved dividing the dataset into k equally sized folds. During each iteration, one fold was designated as the validation set, while the model was trained on the remaining k − 1 folds. This process was repeated k times, with each fold serving as the validation set once. By employing this approach, we obtained a comprehensive evaluation of our predictive model's performance. By calculating the average performance metrics across all iterations, we achieved an extensive assessment of the model's efficacy and its capacity to generalize to unseen data. The adoption of cross-validation served as a safeguard against overfitting, as it offered a more unbiased evaluation of our model's performance. This meticulous technique enhances the reliability of our findings and reinforces the validity of our conclusions. The outcomes of a 5-Folds Cross Validation are presented in Table 5.

**Table 5:** Evaluation results for RF using 5-fold cross validation.

Class	Accuracy	Precision	Recall	F1 score
Fold 1	0.99999	0.99999	0.99999	0.99999
Fold 2	0.99997	0.99997	0.99997	0.99997
Fold 3	0.99999	0.99999	0.99999	0.99999
Fold 4	0.99999	0.99999	0.99999	0.99999
Fold 5	0.99998	0.99998	0.99998	0.99998
Mean	0.99998	0.99998	0.99998	0.99998

**Figure 7:**
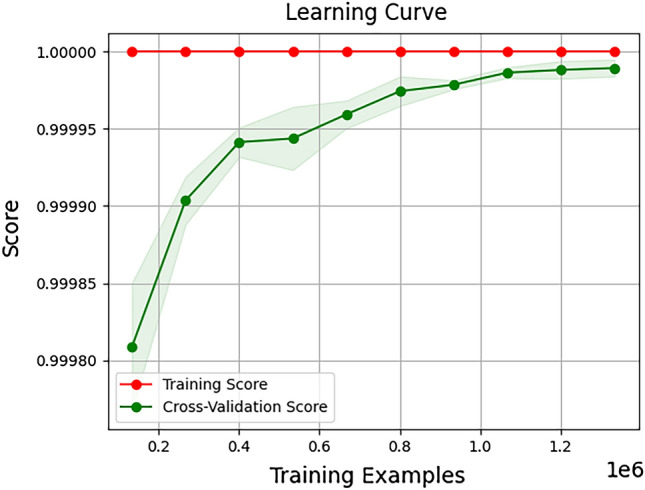
Confusion matrix for RF multi-class classifier.

Figure 7 illustrates the learning curve depicting the performance of the HGB model. The x-axis represents the number of training examples utilized, while the y-axis represents the model's performance. The learning curve consists of two lines: the training score and the cross-validation score. The training score reflects the model's performance on the training data as the number of training examples increases. Conversely, the cross-validation score indicates the model's performance on the validation data during cross-validation. As the number of training examples increases, both the training error and the cross-validation error are expected to improve. The proximity between the two lines indicates the model's capability to generalize. A smaller gap suggests that the model is not excessively fitting the training data and exhibits competent generalization to unseen data.

In order to compare the performance of the proposed ML RF approach with DL based approaches, a pervious study proposed by Zhou et al. ^18^ is used. Their study has been selected as it was the only study that we found during literature review that contains computational analysis. They proposed an intrusion detection model for wireless sensor networks (WSN) based on convolutional neural networks (CNN) and gated recurrent unit (GRU). Their model identifies black hole, gray hole, flooding, scheduling attacks with a reported accuracy of 99.57%. Their experimental platform consisted of an Intel processor with 16 GB memory. For fair comparison, the RF based learning and testing processes were reconducted on CoLab platform using only CPU after removing the GPU accelerator with 12.7 GB memory.

Enhancing the intrusion detection rate of the model can lead to an improvement in the real-time detection performance of the entire IoT intrusion detection system. As shown in Table 6, RF model took 1249.52 s to train, which is less than the spent time for training CNN model. RF model’s temporal complexity as the testing time is 4.33 s. For calculating the average detection time, the testing time is divided on the number of testing dataset instances 4.33/333,559 = 1.298 × 10^−5^ s. This detection time represents only 10% of what is required by CNN.

**Table 6:** Comparison between CNN and RF.

Model	Accuracy	Training set	Training time	Detection time
CNN	0.995700	299,728	2064.10	10.186 × 10^−5^
RF	0.999991	1,334,237	1249.52	1.298 × 10^−5^

## Conclusion and future directions

The paper presented an empirical evaluation for adopting ML tree based algorithms in detecting network intrusions in IoT. Six tree based ML algorithms are implemented and tested using a well-known dataset N-BaIoT for benchmarking. The results demonstrated the significant potential of the tree based ML algorithms. The extensive empirical analysis revealed the superiority of random forest algorithm in compared to the other ensemble trees algorithms for multi-class classification. RF algorithm achieved an accuracy rate of 0.999991 and relative reasonable training and testing times.

Potential future research directions could be focusing on developing comparable models that can identify and thwart new malicious attacks for IoT environment. Especially, addressing further botnet attacks and studying the classification performance in such cases. Taking into account the growing need for comprehensive solutions that can integrate high detection accuracy with light resources consumption.

Another future direction includes evaluating the model on other datasets to investigate the generality. For further an in-depth analysis, a study for the model explainability is required for enhancing transparency and trust.

## Data Availability

The dataset of Median et al. that support the findings of this study is available in Kaggle repository with the identifier [doi: “10.1109/MPRV.2018.03367731”]^45^. For the sake of providing evidence based evaluation, the project along with datasets are uploaded and shared on Kaggle: https://www.kaggle.com/MohamedSaiedEssa/EnsembleTreeBasedIoTNIDS.

## References

[1] “IoT Analytics,” 2023. https://iot-analytics.com/state-of-the-iot-update-q1-q2-2018-number-of-iot-devices-now-7b/ (Accessed Jun. 06, 2023).

[2] Nandy S, Adhikari M, Khan MA (2022). An intrusion detection mechanism for secured IoMT framework based on Swarm-Neural Network. IEEE J. Biomed. Heal. Inform..

[3] Abuhasel KA, Khan MA (2020). A secure industrial internet of things (IIoT) framework for resource management in smart manufacturing. IEEE Access..

[4] Zhang J, Wang Y, Li S, Shi S (2021). An architecture for IoT-enabled smart transportation security system: A geospatial approach. IEEE Internet Things J..

[5] Pandey N, Mishra PK (2023). Detection of DDoS attack in IoT traffic using ensemble machine learning techniques. Netw. Heterog. Media..

[6] Saied M, Guirguis S, Madbouly M (2023). Review of artificial intelligence for enhancing intrusion detection in the internet of things. Eng. Appl. Artif. Intell..

[7] Tian Z, Luo C, Qiu J, Du X, Guizani M (2020). A distributed deep learning system for web attack detection on edge devices. IEEE Trans. Ind. Inform..

[8] Alkadi O, Moustafa N, Turnbull B, Choo KKR (2021). A deep blockchain framework-enabled collaborative intrusion detection for protecting IoT and cloud networks. IEEE Internet Things J..

[9] Qiu J, Tian Z, Du C, Zuo Q, Su S, Fang B (2020). A survey on access control in the age of internet of things. IEEE Internet Things J..

[10] Kotsiantis SB (2013). Decision trees: A recent overview. Artif. Intell. Rev..

[11] Breiman L (2001). Random forests. Mach. Learn..

[12] S. Developers, “Sklearn Ensemble Bagging Classifier.” https://scikit-learn.org/stable/modules/generated/sklearn.ensemble.BaggingClassifier.html (Accessed Apr. 09, 2023).

[13] Freund Y, Schapire RE, Avenue P (1999). A short introduction to boosting. J. Japan. Soc. Artif. Intell..

[14] S. Developers, “Sklearn Ensemble Gradient Boosting Classifier.” https://scikit-learn.org/stable/modules/generated/sklearn.ensemble.GradientBoostingClassifier.html (accessed Apr. 09, 2023).

[15] T. Chen & T. He. xgboost: eXtreme Gradient Boosting. *R Packag. version 0.4-2. 1(4)*, pp. 0–3 (2017).

[16] Pythongeeks, “XGBoost Introduction,” 2022. https://pythongeeks.org/xgboost-introduction/ (accessed Jul. 17, 2023).

[17] Meidan Y (2018). N-BaIoT-Network-based detection of IoT botnet attacks using deep autoencoders. IEEE Pervasive Comput..

[18] Jingjing Z, Tongyu Y, Jilin Z, Guohao Z, Xuefeng L (2022). Intrusion detection model for wireless sensor networks based on MC-GRU. Wirel. Commun. Mob. Comput..

[19] H. Bahsi, S. Nomm, & F. B. La Torre. Dimensionality reduction for machine learning based IoT Botnet Detection. in *2018 15th International Conference on Control, Automation, Robotics and Vision (ICARCV)*, 2018, pp. 1857–1862.

[20] Aloqaily M, Otoum S, Al Ridhawi I, Jararweh Y (2019). An intrusion detection system for connected vehicles in smart cities. Ad Hoc Networks..

[21] Anthi E, Williams L, Słowi M, Theodorakopoulos G, Burnap P (2019). A supervised intrusion detection system for smart home IoT devices. IEEE Internet Things J..

[22] M. Goyal, Ipsit Sahoo, and G. Geethakumari. HTTP botnet detection in IOT devices using network traffic analysis. in *2019 International Conference on Recent Advances in Energy-efficient Computing and Communication (ICRAECC)*, 2019, pp. 1–6.

[23] P. Illy, G. Kaddoum, C. M. Moreira, K. Kaur, & S. Garg. Securing fog-to-things environment using intrusion detection system based on ensemble learning. in *2019 IEEE Wirel. Commun. Netw. Conf.*, pp. 1–7 (2019). 10.1109/WCNC.2019.8885534.

[24] “NSL-KDD dataset.” https://www.unb.ca/cic/datasets/nsl.html (accessed Jul. 30, 2023).

[25] Alsulami AA, Al-haija QA, Tayeb A, Alqahtani A (2022). An intrusion detection and classification system for iot traffic with improved data engineering. Appl. Sci..

[26] I. Ullah & Q. H. Mahmoud. A scheme for generating a dataset for anomalous activity detection in IoT networks a scheme for generating a dataset for anomalous activity detection in IoT. in *Canadian Conference on Artificial Intelligence (CCAI), Ottawa, ON, Canada,* 2020, no. April 2021, pp. 508–520. 10.1007/978-3-030-47358-7.

[27] P. Chaudhary & B. B. Gupta. DDoS detection framework in resource constrained internet of things domain. in *2019 IEEE 8th Glob. Conf. Consum. Electron. GCCE 2019*, pp. 675–678 (2019). 10.1109/GCCE46687.2019.9015465.

[28] Manimurugan S, Al-mutairi S, Aborokbah M, Chilamkurti N, Ganesan S, Patan R (2020). Effective attack detection in internet of medical things smart environment using a deep belief neural network. IEEE Access.

[29] Stiawan D, Yazid M, Bamhdi AM (2020). CICIDS-2017 dataset feature analysis with information gain for anomaly detection. IEEE Access.

[30] J. Alsamiri & K. Alsubhi. Internet of things cyber attacks detection using machine learning. *Int. J. Adv. Comput. Sci. Appl.* **10**(12), 627−634 (2019).

[31] Koroniotis N, Moustafa N, Sitnikova E, Turnbull B (2019). Towards the development of realistic botnet dataset in the internet of things for network forensic analytics: Bot-IoT dataset. Futur. Gener. Comput. Syst..

[32] R. Doshi, N. Apthorpe, & N. Feamster. Machine learning DDoS detection for consumer internet of things devices. in *Deep Learning and Security Workshop (DLS). IEEE*, 2017, no. Ml.

[33] O. P. Dwyer, A. K. Marnerides, V. Giotsas, & T. Mursch. Profiling IoT-based Botnet Traffic using DNS. in *IEEE global communications conference (GLOBECOM)*, pp. 1–6 (2018).

[34] Hasan M, Islam MM, Zarif MII, Hashem MMA (2019). Attack and anomaly detection in IoT sensors in IoT sites using machine learning approaches. Internet Things Netherlands..

[35] F.-X. A. M.-O. Pahl. DS2OS traffic traces. (2018). https://www.kaggle.com/datasets/francoisxa/ds2ostraffictraces (accessed Jun. 20, 2023).

[36] I. Alrashdi, A. Alqazzaz, E. Aloufi, R. Alharthi, M. Zohdy, & H. Ming. AD-IoT: Anomaly detection of IoT cyberattacks in smart city using machine learning. in *2019 IEEE 9th Annu. Comput. Commun. Work. Conf.*, pp. 305–310 (2019). 10.1109/CCWC.2019.8666450.

[37] Moustafa N, Slay J (2015). UNSW-NB15: A comprehensive data set for network intrusion detection systems (UNSW-NB15 network data set). Military Commun. Inform. Syst. Conf. (MilCIS)..

[38] Thamilarasu G, Odesile A, Hoang A (2020). An intrusion detection system for internet of medical things. IEEE Access..

[39] Eskandari M, Janjua ZH, Vecchio M, Antonelli F (2020). Passban IDS: An intelligent anomaly based intrusion detection system for IoT edge Devices. IEEE Internet Things J..

[40] Hammoudeh M, Aljaberi SM (2021). Modeling of deep learning based intrusion detection system in internet of things environment. J. Cybersecurity Inf. Manag..

[41] Al Tobi AM, Duncan I (2018). KDD 1999 generation faults: A review and analysis. J. Cyber Secur. Technol..

[42] M. Saied & S. Guirguis. Evaluation of tree based machine learning algorithms for network intrusion detection in IoT. in *IEEE IT Prof.* (2023).

[43] M. Saied, S. Guirguis, & M. Madbouly. A comparative study of using boosting-based machine learning algorithms for IoT network intrusion detection. *Int. J. Comput. Intell. Syst.* **16**(1), 1–15 (2023).

[44] M. Alqahtani, H. Mathkour, & M. M. Ben Ismail. IoT botnet attack detection based on optimized extreme gradient boosting and feature selection. *Sensors* **20**(21), 1–21 (2020). 10.3390/s20216336 PMC7664261 33172023

[45] K. Naveed, H. Wu, & A. Abusaq. Dytokinesis : A cytokinesis-inspired anomaly detection technique for IoT devices. in *IEEE 45th Conference on Local Computer Networks*, pp. 373–376 (2020).

[46] Al-Haija QA, Al-Badawi A, Bojja GR (2021). Boost-defence for resilient IoT networks: A head-to-toe approach. Expert Syst..

[47] Alsaedi A, Moustafa N, Tari Z, Mahmood A, Anwar A (2020). TON_IoT telemetry dataset: A new generation dataset of IoT and IIoT for data-driven intrusion detection systems. IEEE Access..

[48] Al-Haija QA, Al-Dalaien M (2022). ELBA-IoT: An ensemble learning model for botnet attack detection in IoT networks. Sensors Actuator Netw..

[49] Almiani M, Abughazleh A, Al-rahayfeh A, Atiewi S, Razaque A (2019). Deep recurrent neural network for IoT intrusion detection system. Simul. Model. Pract. Theory..

[50] R. Panigrahi & S. Borah. A detailed analysis of CICIDS2017 dataset for designing Intrusion Detection Systems. *Int. J. Eng. Technol.* **7**(3), 479–482 (2018).

[51] Ashmore R, Calinescu R, Paterson C (2021). Assuring the machine learning lifecycle: Desiderata, methods, and challenges. ACMComputing Surv..

